# Maternal Deprivation Induces Memory Deficits That Are Reduced by One Aerobic Exercise Shot Performed after the Learning Session

**DOI:** 10.1155/2019/3608502

**Published:** 2019-11-16

**Authors:** Priscila Marques Sosa, Ben-Hur S. Neves, Guilherme Salgado Carrazoni, Gabriela Mendes Gomes, Gabriel Del Rosso, Bruna Piaia Ramborger, Rafael Rohers, Pâmela Billig Mello-Carpes

**Affiliations:** ^1^Physiology Research Group, Stress, Memory and Behavior Lab, Federal University of Pampa, Uruguaiana, RS, Brazil; ^2^Grupo Interdisciplinar de Pesquisa em Prática de Ensino, Universidade Federal do Pampa, Uruguaiana, RS, Brazil

## Abstract

During the neonatal period, the brain is susceptible to external influences. Exposure to stressful events during this phase of life influences brain development and impacts adult life. In animals, the maternal deprivation (MD) model is effective in mimicking stress in the early stages of development. In contrast, physical exercise seems to be able to prevent deficits in memory consolidation. Although the effects of chronic exercise in cognition are already well established, little is known about the effects of acute aerobic exercise. Here, male Wistar rats divided into deprived (MD) and nondeprived (NMD) rats were submitted to the object recognition (OR) memory test. Immediately after OR training, some of the rats were submitted to a single aerobic exercise session for 30 minutes. Memory consolidation and persistence were evaluated by retention tests performed 24 h and 7, 14, and 21 days after OR training. We show that a single physical exercise session is able to modulate learning by promoting memory consolidation and persistence in rats with cognitive deficits induced by MD. Hippocampal dopamine levels, measured by HPLC, were not altered after OR training in rats that performed and in rats that did not perform an exercise session; on the other hand, while OR training promoted increase of hippocampal norepinephrine in NMD rats, the MD rats did not present this increase, regardless of the practice or not of exercise.

## 1. Introduction

Exposure to stressful environmental events during early childhood may impact neural plasticity and increase the vulnerability to psychopathologies [1]. The early life stress causes neuroendocrine changes associated with social and emotional behavior alterations [2] that may persist throughout life [3]. In humans, especially from the prenatal period until the early years of postnatal life, the brain undergoes a rapid development [4] and is highly sensitive to the influence of positive and negative external experiences [5].

An example of early childhood stress is the maternal separation; in humans, it can be related to institutionalization in orphanages [6], which has already been shown to be responsible for long-term behavioral damages, such as increased anxiety [7], and psychopathologies, as schizophrenia [8]. Several reports suggest that early childhood traumas may affect distinct neural circuits, such as those involving the dopaminergic and the noradrenergic systems [9–13], which perform several functions in the brain, including the regulation of cognition, attention, and stress [12, 14–17]. In addition, neuroimaging studies in humans suggest that early childhood stress interferes in the maturation of the dopaminergic system [18].

In rodents, maternal deprivation (MD) is a stress model widely used as a paradigm to study early life adverse events [19], as parental abuse or loss [11]. In this model, there is evidence that early-stage stress leads to changes in dopamine neurotransmission, affecting both dopamine (DA) levels [20] and dopaminergic receptor density (D1 and D2) [18]. In addition to interactions with the dopaminergic system, evidences suggest that early childhood stress also changes noradrenergic neural networks such as the locus-coeruleus-norepinephrine (NE) system [21, 22].

In contrast, some strategies have been used to improve brain DA and/or NE levels, as well as cognitive functions. One is the physical exercise. Chronic aerobic exercise effects on cognition are widely studied [23, 24], and researches demonstrated that exercise improves behavioral, mental health [25, 26] and biochemical parameters [23, 27, 28]. Despite other mechanisms involved on the aerobic exercise effects, it acts by exciting the central nervous system through positive regulation of catecholamines, including epinephrine (E), NE, and DA [29–32]. Furthermore, a previous study from our laboratory demonstrated that a single exercise session is able to promote object recognition memory persistence, which is related to the increase of NE levels [33].

Thus, in the present work, we aim to investigate the effects of a single aerobic exercise session on memory deficits related to MD in rats. Additionally, we investigated the impact of MD and a single aerobic exercise session on hippocampal DA and NE levels.

## 2. Materials and Methods

### 2.1. Animals and Experimental Design

Pregnant female Wistar rats were obtained from the Central Vivarium of the Federal University of Santa Maria (RS/Brazil). All animals were kept in a light/dark cycle of 12 h (7:00 a.m. to 7:00 p.m.) at controlled ambient temperature (23 ± 2°C) and air humidity (60 ± 5%). The rats were housed individually, with food and water *ad libitum*.

The delivery day was considered as day zero. From the postnatal day 1 (PND-1) to the PDN-10, the MD protocol was performed with half of the pups, as described below. The animals were weaned in PND-21 and housed 5 per cage. Only males were used in the experiments described in this paper. All experiments were performed according to the principles of laboratory animal care (NIH Publication No. 80-23, revised 1996) and approved by the Institutional Committee on Animal Care and Use of the Federal University of Pampa (RS/Brazil) (050/2017).

The rats were divided into six groups, according the procedures adopted, which are described below.

- *NMD*. Control rats were not submitted to the MD protocol but were trained in the object recognition memory (OR) task (*n* = 15)
- *MD*. The rats were submitted to the MD protocol and were trained in the OR task (*n* = 15)
- *NMD+FAE*. The rats were not submitted to the MD protocol, were familiarized to the exercise apparatus (a treadmill for rodents), and were trained in the OR task, without the exercise shot after OR training (*n* = 15)
- *MD+FAE*. The rats were submitted to the MD protocol, were familiarized to the exercise apparatus, and were trained in the OR task, without the exercise shot after OR training (*n* = 15)
- *NMD+AE*. The rats were not submitted to the MD protocol, were familiarized to the exercise apparatus, were trained in the OR task, and were submitted to an aerobic physical exercise shot immediately after (*n* = 15)
- *MD+AE*. The rats were submitted to the MD protocol, were familiarized to the exercise apparatus, were trained in the OR, and were submitted to an aerobic physical exercise shot immediately after OR training (*n* = 15)

**Figure 1:**
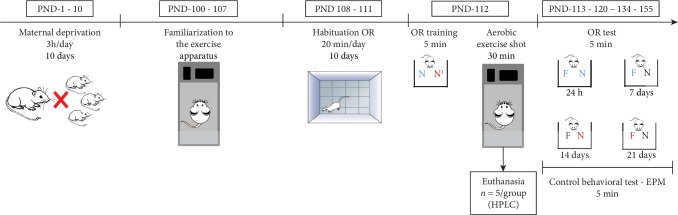
Experimental design. Half of the animals were submitted to the maternal deprivation (MD) protocol from PND-1 to PND-10 (groups ii, iv, and vi). Animals from groups iii to vi were familiarized to the physical exercise apparatus from PND 100 to PND 107. All animals, except the naïve, were habituated to the object recognition (OR) memory task apparatus (PND 108-111) and trained in the OR task (PND112), when they were exposed to two different new (N) objects for free exploration. Animals of groups v and vi were submitted to an aerobic exercise shot immediately after OR training. After the aerobic exercise shot, 5 animals from each group, plus 4 naïve animals, were euthanized for biochemical analysis (HPLC). The other animals were submitted to OR tests 24 h and 7, 14, and 21 days after the OR training (PNDs 113, 120, 134, and 155), when the animals were exposed to a familiar (F) and to a new object (N) for free exploration. On each testing day, always after the OR test session, the Elevated Plus Maze (EPM) test was performed as a control behavioral test to verify anxiety.

After, ten rats from each group were submitted to OR memory testing 24 h and 7, 14, and 21 days after OR training (Figure 1). In all testing days, the Elevated Plus Maze task was performed after OR testing, as an anxiety control test. Five animals from each group were euthanized immediately after the exercise shot, their brains were isolated, and the hippocampi were quickly dissected for use in biochemical tests. For biochemical tests, additional naïve rats were used (*n* = 4); these animals were not submitted to any behavioral procedure.

### 2.2. Maternal Deprivation

Female Wistar rats were kept in individual boxes until the delivery day (day 0). The rats of groups ii, iv, and vi were submitted to maternal deprivation (MD) for 10 days (PND-1 to PND-10), 3 hours/day, always during the light part of the light/dark cycle.

The MD protocol consisted in removing the mother from the residence box to another room, without touching the puppies. Pups were kept in their cage, and while the mothers were absent, the room temperature was maintained at around 32°C to compensate the loss of the mother's body heat [34]. At the end of each daily deprivation session, the mothers were returned to their original boxes.

The rats of groups i, iii, and v remained in their residence boxes with their mothers until PND-21. On PND-21, all rats were weaned, and males were kept in groups of 5 in plastic boxes, similar to all other animals in our accommodation facility. The female pups were donned to other experiments. This specific MD protocol was selected since in previous studies no changes in anxiety or other behavioral parameters that could interfere with the memory testing were found [34–36].

### 2.3. Familiarization with the Exercise Apparatus

Prior to the memory task, rats were familiarized with the treadmill where the physical exercise would be performed to avoid novelty and/or stress effects. Initially, the rats were familiarized with the treadmill for three days (first day static and then with treadmill at a speed of 2 to 5 m/min for 10 min).

Afterwards, they were submitted to the “good runner protocol,” which consists in placing the animals on a treadmill without slope for three consecutive days (velocity of 8 m/min for 10 min) and then assessing the level of trainability, considering a scale from 1 to 5 points (1: refuses to run; 2: runners below average (runs and stops or runs in the wrong direction); 3: average runner; and 4: above average (runs well), with sporadic stops on the treadmill); in the end, rats that maintained a mean of three or more points were included in the exercise groups [37].

On the last day, an indirect VO_2_ maximum test was performed to determine individual exercise intensity (starting at a slow rate increased by 5 m/min every 3 minutes until the rat could not continue to run) [38, 39].

### 2.4. Habituation and Training in the Object Recognition Memory Task

The object recognition (OR) training and tests were performed in an arena (50 × 50 × 50 cm) constructed of polyvinyl chloride, plywood, and transparent acrylic, as described by Ennaceur and Delacour [40]. The sessions were filmed for further analyses, and the experimenters were blinded to animals' experimental condition. Firstly, the animals were familiarized to the OR apparatus by placing them inside the arena for 20 min per day to freely explore on 4 consecutive days prior to OR training. On the training day, two different objects (N and N^1^), equally attractive for the rats, were placed in the apparatus and the animals were free to explore them for 5 min. The objects were made of metal, glass, or glazed pottery. The time exploring each object was accounted.

### 2.5. Aerobic Exercise Shot

Animals from groups v and vi were submitted to a single physical exercise session immediately after the OR training, using a treadmill built for rodents (Insight Ltda., São Paulo, Brazil). The protocol used was adapted from Malek et al. [41]; an intensity of 60-70% of maximum oxygen consumption (VO_2_) in a single session for 30 min was used.

### 2.6. Object Recognition Memory Tests

Twenty-four hours and 7, 14, and 21 days after training, in the memory testing phase, one of the objects used in OR training was maintained (Familiar object—F) and the other was randomly replaced by a new one (N). In each test day, the objects were placed in the apparatus and the animals were free to explore them for 5 min. The test sessions were recorded, and after, the time exploring each object was accounted.

### 2.7. Elevated Plus Maze (EPM) Test

To assess the anxiety state, which could affect the results of memory tests, the rats were exposed to EPM in each testing days, always after the OR test. The EPM apparatus consists of a raised platform, located 60 cm above the ground, and has two closed arms face to face and two open arms, each measuring 50 × 10 cm, containing infrared sensors. The closed arms are composed of sidewalls 20 cm high. The time spent and the total number of entries in the open and closed arms were recorded in a 5-minute session [42].

### 2.8. Biochemical Testing

Five rats from groups i to vi were sacrificed immediately after the single session of aerobic exercise or in the equivalent time. Additionally, 4 naïve groups were sacrificed. The rats' brains were quickly removed for dissection of the bilateral hippocampus. The tissues were homogenized in 50 mM HCl, pH 7.4 (1/5, *w*/*v*). Subsequently, the samples were centrifuged at 2400 g for 10 min, and the supernatants (S1) were filtered and then stored at -80°C for further analysis.

The levels of DA and NE in hippocampus homogenates were determined using an “Inertsil ODS-3” reverse-phase High Performance Liquid Chromatography (HPLC) system (5 *μ*4.6 × 250 mm, GL Sciences) with a diode array detector as described by [43]. The mobile phase consisted of methanol and water (12/88, *v*/*v*) adjusted to pH 3 with phosphoric acid. The concentrations of the neurotransmitters relative to the standard analytical curve equation were obtained and corrected according to the dilution and sample weight.

To separate DA and NE, we used the isocratic programming with a flow rate of 0.8 mL/min. The sample was filtered through 0.22 *μ*m syringe filters. We inject 20 *μ*L of samples into the HPLC system by an automatic sampling device (YL9150). Detection was at 198 nm by DAD. Chromatograms were recorded and integrated by PC integration software (YLClarity). All analyses were performed in triplicate. Analytical parameters were as follows: linear range, 0.1-10.0 *μ*g/mL; coefficient of determination, 0.999; and calibration equation, *y* = 628,12*x*‐34,342. DA and NE for HPLC were supplied by Sigma-Aldrich, Brazil. Other reagents used in this experiment were from analytical grades and obtained from standard commercial suppliers.

### 2.9. Statistical Analyses

The data distribution was analyzed using the Shapiro-Wilk test.

The exploration time of the objects in the OR task was converted to a percentage of the total exploration time on the training or test sessions, and the Wilcoxon signed-rank test was used to compare the percentage of the total exploration time spent in each object considering a theoretical mean of 50%.

The EPM data were compared by the Kruskal-Wallis test.

The HPLC results and total exploration time in each OR session were compared by ANOVA, followed by Tukey's multiple comparison tests.

The results are expressed as the mean ± SD. In all analyses, *P* values < 0.05 were considered significant.

## 3. Results

### 3.1. Object Recognition Memory Consolidation and Persistence

**Figure 2:**
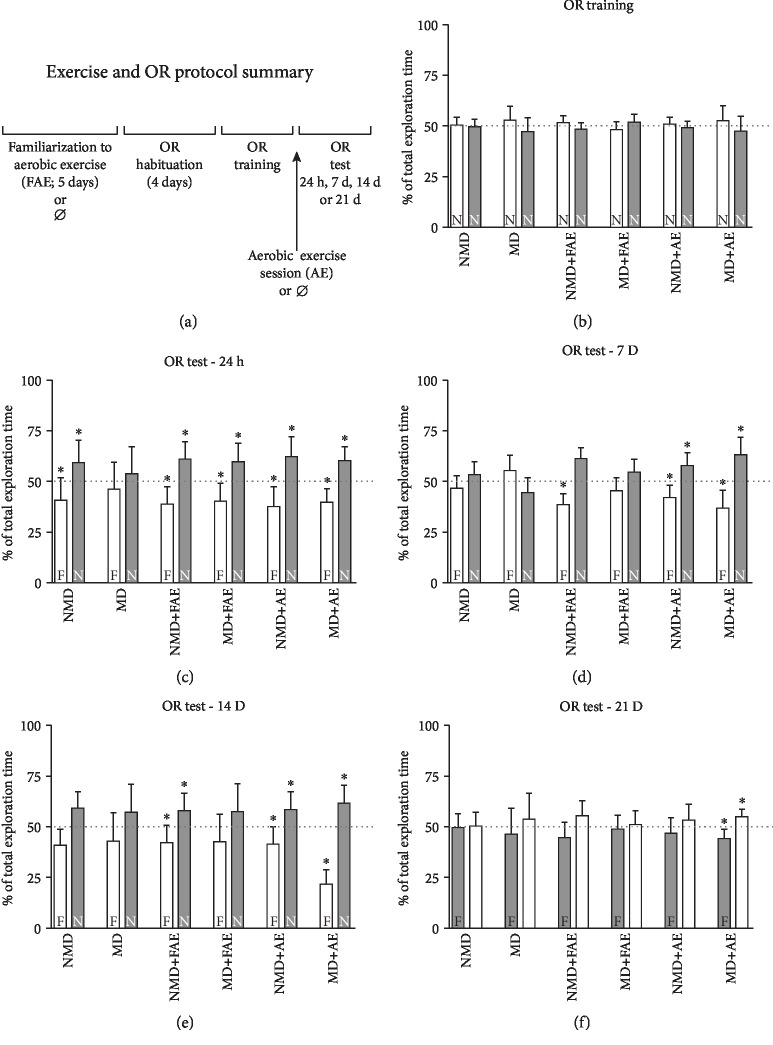
Maternal deprivation (MD) causes long-term memory deficits of object recognition (OR) memory. An acute aerobic exercise session is able to reverse these deficits. (a) The animals were trained in the OR task and tested 24 hours and 7, 14, and 21 days after; animals from some groups were submitted to familiarization of aerobic exercise in a treadmill (NMD+FAE, MD+FAE, NMD+AE, and MD+AE), and animals from some groups were submitted to an aerobic exercise session just after the OR training session (NMD+AE and MD+AE). (b) In the training session, the animals were exposed to two new objects (N), and animals from all groups explored about 50% of the total exploration time each one. (c) In the OR consolidation memory test (24 h), the rats were exposed to a familiar (F) and to a new object (N). The MD group was not able to distinguish the familiar from the new object in the test session but the MD rats familiarized to treadmill (FAE) and submitted to a shot of physical exercise (AE) were. When tested 7 (d) and 14 (e) days after training, neither the MD group nor the NMD group was able to distinguish the familiar and new objects. All rats that performed acute physical exercise were able to distinguish the familiar object from the new one. (f) After 21 days, only MD rats submitted to exercise present persistence of memory. The data are expressed as the mean ± standard deviation of the percentage of total exploration time; ^∗^*P* < 0.05 for the Wilcoxon signed-rank test, considering a theoretical mean of 50%; *n* = 8‐10 per group. OR = object recognition test; NMD = nonmaternal-deprived rats; MD = maternal-deprived rats; NMD+FAE = nonmaternal-deprived rats submitted to aerobic exercise familiarization; MD+FAE = maternal-deprived rats submitted to aerobic exercise familiarization; NMD+AE = nonmaternal-deprived rats submitted to aerobic exercise familiarization and to an aerobic exercise session; MD+AE = maternal-deprived rats submitted to aerobic exercise familiarization and to an aerobic exercise session.

Memory consolidation and persistence were assessed by the OR test. During OR training, the rats from all groups explored the two objects by a similar percentage of total exploration time, ~50% of the total exploration time each one (*P* = 0.704, *t*_(8)_ = 0.393 for NMD group; *P* = 0.242, *t*_(9)_ = 1.288 for MD group; *P* = 0.155, *t*_(9)_ = 1.552 for NMD+FAE group; *P* = 0.190, *t*_(9)_ = 1.416 for MD+FAE group; *P* = 0.460, *t*_(7)_ = 0.780 for NMD+AE group; and *P* = 0.310, *t*_(9)_ = 1.075 for MD+AE group; Figure 2(b)).

In the 24 h test, the control group (NMD) explored significantly more than 50% of the new object (*P* = 0.039, *t*_(8)_ = 2.513; Figure 2(c)). In persistence tests, this difference was not observed (for 7: *P* = 0.250, *t*_(8)_ = 1.718, Figure 2(d), NMD; for 14: *P* = 0.062, *t*_(6)_ = 0.038 exploring the familiar object in relation to the new object, Figure 2(e), NMD; and for 21 days: *P* = 0.734, *t*_(9)_ = 0.168, Figure 2(f), NMD).

As expected, MD rats did not consolidate memory. On the 24 h test, they explored ~50% of the total exploration time each object (*P* = 0.570, *t*_(9)_ = 0.8847; Figure 2(c), MD), which is observed until the 21-day test (*P* = 0.125, *t*_(7)_ = 1.84 for 7-day test, Figure 2(d), MD; *P* = 0.082, *t*_(9)_ = 1.972 for 14-day test, Figure 2(e), MD; and *P* = 0.406, *t*_(9)_ = 0.916 for 21-day test, Figure 2(f), MD).

Nondeprived rats (NMD) familiarized to the exercise apparatus (NMD+FAE) explored more than ~50% of the new object in comparison to the familiar one in the 24 h test (*P* = 0.002, *t*_(9)_ = 4.239, Figure 2(c), NMD+FAE). The memory persisted up to 14 days after the training (*P* = 0.002, *t*_(9)_ = 6.663 for 7-day test, Figure 2(d), NMD+FAE; *P* = 0.037, *t*_(9)_ = 2.868 for 14-day test, Figure 2(e), NMD+FAE). Memory persistence was not observed on the 21-day test (*P* = 0.064, *t*_(9)_ = 2.245, Figure 2(f), NMD+FAE).

The MD rats familiarized to the exercise apparatus (MD+FAE) explored significantly more than 50% of the new object in the 24 h test (*P* = 0.019, *t*_(9)_ = 3.402; Figure 2(c), MD+FAE) but not in the memory persistence tests (*P* = 0.05, *t*_(9)_ = 2.255 for the 7-day test, Figure 2(d), MD+FAE; *P* = 0.160, *t*_(9)_ = 1.724 for the 14-day test, Figure 2(e), MD+FAE; and *P* = 0.652, *t*_(9)_ = 0.507 for the 21-day test, Figure 2(f), MD+FAE).

Nondeprived rats who performed aerobic exercise on the treadmill (NMD+AE) explored significantly more than ~50% of the new object in the 24 h test (*P* = 0.015, *t*_(7)_ = 3.429, Figure 2(c), NMD+AE). Memory persistence was observed until 14 days after training (*P* = 0.015, *t*_(7)_ = 3.617 for 7-day test, Figure 2(d), NMD+AE; *P* = 0.031, *t*_(7)_ = 2.784 for 14-day test, Figure 2(d), NMD+AE). On the test performed 21 days after training, memory persistence was not observed (*P* = 0.312, *t*_(7)_ = 1.215, Figure 2(f), NMD+AE).

The maternal-deprived rats submitted to physical exercise shot (MD+AE) presented a good memory consolidation, since they explored the new object for more than 50% of the total exploration time in the 24 h test (*P* = 0.002, *t*_(9)_ = 4.774; Figure 2(c), MD+AE). In addition, the memory persisted for 21 days (*P* = 0.007, *t*_(8)_ = 4.429 for 7-day test, Figure 2(d), MD+AE; *P* = 0.003, *t*_(9)_ = 4.140 for 14-day test, Figure 2(e), MD+AE; and *P* = 0.003, *t*_(9)_ = 4.057 for 21-day test, Figure 2(f), MD+AE).

It is importantly highlighted that despite the differences between groups in the percent of time spent exploring the new and the familiar objects in some testing sessions, no differences in the total exploration time were found between the groups in any OR session (Table 1).

### 3.2. Elevated Plus Maze

MD and aerobic physical exercise protocol did not alter the anxiety of the animals in the testing session performed 24 h and 7, 14, and 21 days after training (Table 2).

### 3.3. Catecholamine Levels

**Figure 3:**
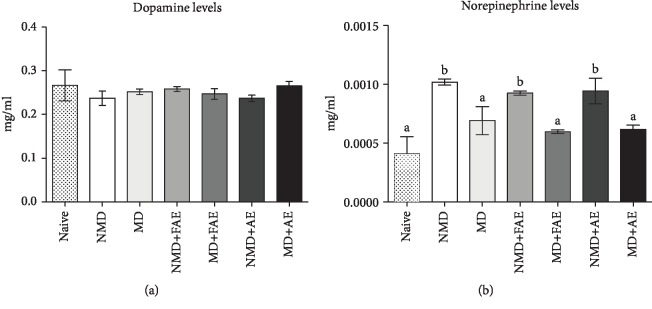
Hippocampal dopamine and norepinephrine levels on nonmaternal-deprived and maternal-deprived rats. Rats were trained in the OR task; some of them were submitted to an aerobic exercise shot, and after, hippocampus was removed. Bars depict HPLC dopamine (a) and norepinephrine (b) levels in hippocampal homogenates (mean ± SD). All bars with letter “A” are statistically equal and differ significantly to all bars with letter “B.” *P* ≤ 0.05 on ANOVA followed by Tukey's multiple comparison test; *n* = 3‐5 per group, analyzed in triplicate. OR = object recognition test; NMD = nonmaternal-deprived rats; MD = maternal-deprived rats; NMD+FAE = nonmaternal-deprived rats submitted to aerobic exercise familiarization; MD+FAE = maternal-deprived rats submitted to aerobic exercise familiarization; NMD+AE = nonmaternal-deprived rats submitted to aerobic exercise familiarization and to an aerobic exercise session; MD+AE = maternal-deprived rats submitted to aerobic exercise familiarization and to an aerobic exercise session.

No differences were found between groups on hippocampal dopamine levels (*F*_(6, 95)_ = 1.33; *P* = 0.2513, Figure 3(a)). On the other hand, differences were found on hippocampal norepinephrine levels between groups (*F*_(6, 54)_ = 13.08; *P* < 0.0001, Figure 3(b)): NMD groups presented higher NE levels than naïve animals (*P* < 0.001). MD groups presented lower NE levels than NMD groups (*P* < 0.05 for all comparisons, Figure 3(b)). There are no differences between MD and MD+FAE groups (*P* > 0.05, Figure 3(b)) or MD+AE group (*P* > 0.05, Figure 3(b)).

## 4. Discussion

It is well established that the exposure to stress in the postnatal period can trigger numerous effects on neuroplasticity, cognitive function, and behavior in adulthood [44], since the postnatal period is critical for the central nervous system development [4]. In this sense, strategies that seek to minimize and/or reverse cognitive damages related to this type of stress are important. Here, we investigate the effects of an aerobic exercise (AE) shot on the consolidation and persistence of object recognition (OR) memory in a MD rat model. Our results showed that a single shot of exercise is able to modulate learning, promoting the acquisition of memory in MD rats, as well as the persistence of this memory up to 21 days. Additionally, the session of AE is able to improve the memory of NMD rats, since the memory persists for more time in these rats than in the ones that do not performed exercise. Still, the simple previous familiarization to the physical exercise apparatus to OR training promoted an improvement on learning.

Our results show that control animals (NMD) do not present memory persistence beyond 24 hours. It is importantly emphasized that it was expected, since this type of memory trace generally is susceptible to physiological decay [33]. On the other hand, MD rats are not able to learn, as previously demonstrated [34, 36, 45]. Aerobic exercise (AE) was able to revert MD memory deficits, and MD+AE rats present better results than the NMD group in 7, 14, and 21 days because in control (NMD) rats, i.e., normal rats that were not submitted to any procedure despite the memory task, the OR memory trace normally decays along time. A single session of acute exercise, on the other hand, is aimed at modulating OR memory consolidation, and, as in NMD animals submitted to exercise, the MD animals that performed exercise had an improvement in consolidation with consequent improvement in the persistence of this memory. These results are associated with the changes in brain activity after exercise [33], showing that improved memory consolidation may result in improved memory persistence [46]. This explains why MD+AE animals show better memory than the NMD+FAE group, since these animals were not submitted to exercise after learning; so, memory consolidation was not modulated, and these animals show memory consolidation but did not show the same memory persistence (NMD+FAE). On the other hand, the MD+AE animals were the only that presented memory persistence until 21 days. Initially, we are expecting that the NMD+AE also presented this effect on memory, but it was not observed. We hypothesized that the brain changes induced by MD may make the brain of these animals more susceptible to changes after external influences, such as the practice of an exercise session [1]. One possible explanation to this result is that the neural changes induced by MD [1] may make the brain of these animals more susceptible to external influences along life or make them respond in a different way; so, factors as the practice of an exercise session could impact in a more expressive way the MD animals than control (NMD) ones.

Physical exercise is a noninvasive and easy alternative to influence the cognitive functions. Previous studies of our laboratory demonstrated that chronic aerobic exercise minimizes mnemonic deficits in animal models of neurodegenerative diseases [24, 36, 47], which have been related to antioxidant and anti-inflammatory exercise effects [23, 27], besides the exercise influence on neuroplasticity [28]. However, acute aerobic exercise is still studied little as a neuroprotective strategy [48].

In a recent study, we demonstrated that normal rats submitted to one acute aerobic exercise session (30 minutes) after the OR learning present memory persistence [33]. The memory persistence induced by exercise was associated with the increase of NE levels in the hippocampus induced by exercise [33], since this neurotransmitter involvement on memory persistence is recognized [49]. Here, we confirmed that the aerobic exercise shot is able to improve memory, promoting its persistence, as previously reported [33], and, for the first time, we show that one exercise session can minimize the memory deficits related to maternal deprivation. MD rats that were not able to consolidate OR memory, when submitted to one session of exercise after OR learning, not only consolidate memory but also form a strong memory, which persists for 21 days, at least.

It is important to consider that our results show that the simple familiarization to the exercise apparatus had some effects on memory. Familiarization to exercise was able to improve memory persistence in NMD animals—at 7 and 14 days, but not at 21, the same result of NMD+AE animals. Also, familiarization improved memory retention in MD animals (24 h test), which is not observed on MD animals not exposed to treadmill familiarization. This result could be attributed to a persistent effect of novelty (the physical exercise apparatus is a novelty for the rats), which has been shown to enhance short- and long-term memory by protein synthesis induction [45] or, what we consider more likely, to the familiarization to the treadmill, which could be considered as a short-term physical training and could induce some recognized physical exercise effects on the brain, as neuroplasticity, increasing brain blood supply, and others [23, 24, 27, 28, 36, 47]. Initially, we included the familiarization group because the exercise group needed to be submitted to the familiarization to treadmill before the exercise session, anyway the animals would have difficulties on running, and they could be stressed, so we would like to guarantee that this procedure did not have effects per se. What we found is that aerobic exercise presented better effects than familiarization, especially on rats with memory deficit related to MD, but familiarization also promoted benefits to memory.

The physiological mechanisms involved in the effects of an acute aerobic exercise can be many; among them, it is known that exercise can promote increase of catecholamine levels as norepinephrine (NE) and dopamine (DA) [50], neurotransmitters involved on modulation of memory consolidation. In fact, we observed that all NMD groups showed increase of NE levels in comparison to the naïve group. However, we were not able to verify the same changes in hippocampal NE levels after OR training in MD rats or in the rats submitted to AE session. On the other hand, differences on DA levels were not observed between groups.

A previous study showed that MD causes a decrease in tyrosine hydroxylase enzyme in rodents [51], which is the first enzyme for dopaminergic metabolism, converting tyrosine into dopamine precursor DOPA [52]. Considering this finding, we could think that a decrease in NE levels in MD rats may be due to a gap in the enzyme; however, a decrease in tyrosine hydroxylase enzyme would also lead to a decrease in DA levels, which we could not demonstrate in this study. On the other hand, it is known that the main source of NE to the hippocampus is the locus coeruleus (LC) [21] a structure located in the brain stem, whose neurons are affected by MD. MD affects the dendritic morphology of the LC neurons [22], which may impair noradrenergic synapses in the hippocampus and could explain the impossibility to improve NE release in the hippocampus after OR training. This decrease of NE on MD rat hippocampus was not reversed by acute exercise, since one single physical exercise session is not able to promote immediate protein synthesis and neuroplasticity that would be required to revert this morphological alterations [53].

Moreover, the difference between DA and NE levels observed might be due to the incoordination between the control of noradrenergic systems, which is influenced by sympathoadrenal and central nervous systems, and dopaminergic, influenced exclusively by the central nervous system [54] previous works on MD models highlighted the impact of this intervention on the pituitary-adrenal axis, which could influence specially NE sources [55]. Corroborating to our work, a previous study with adult female rats showed that in the face of different MD protocols there were no significant differences in DA levels [56]; the authors highlighted that the duration of stress exposure and the time elapsed between stress exposure and euthanasia may influence these inconsistent findings. Another possible explanation for the discrepancy between this study and the others that find dopaminergic alterations on MD rats is the variation in age, as most studies use MD models in adolescent rats and we used young adults [18, 20, 56].

Surprisingly, physical exercise did not promote NE alterations, as expected. These results also contradict previous results, although some of them use a single exercise session performed at high intensity without prior habituation, i.e., a protocol that differs a little from ours [31, 57]. This result suggests that increasing catecholamine levels could be related to the exercise intensity. Nonetheless, we can affirm that our results are specifically related to exercise effects, since, contrary to other studies, we did not use any type of aversive stimulus prior to or during the exercise session [58].

Despite the fact that we did not find significant differences in DA levels, we cannot assume that MD did not cause any disturbance on the dopaminergic system, as well as that the exercise did not influence this neurotransmitter release. It is important to highlight that the technique used can influence the measurements of DA and NE [59]. Although the HPLC technique used in our study had been previously used [33, 43], it is important to consider that the degradation of DA and NE can occur from 200 to 2000 milliseconds [52], which made their measurement difficult. So, measurements by *in vivo* microanalysis may provide more reliable results [60].

Finally, the improvement of cognitive deficits promoted by acute aerobic exercise may be associated with other factors that do not necessarily involve catecholamine modulation. Recent studies indicate that a single acute aerobic exercise session is effective to increase BDNF (brain-derived neurotrophic factor) mRNA, even in the absence of noradrenergic signaling [58]. BDNF is an important protein related to neural plasticity and closely related to long-lasting memory consolidation [61]. In addition, other studies suggest that epigenetic modulation through acute aerobic exercise [62] may induce histone acetylation, increasing HAT activity (histone acetyltransferases) in combination with the decrease of the activity of HDAC (histone deacetylases) in rat hippocampus, leading to neuroprotective effects [63].

In summary, acute aerobic exercise is an important procognitive stimulus that acts to attenuate cognitive deficits caused by MD. Despite the fact that we are not able to determine the exact modulatory mechanisms involved in the acute exercise effects, it is clearly an additional valuable measure to sustain the cognitive functions caused by early life stress.

## 5. Conclusion

MD promotes object recognition (OR) memory deficits. Acute physical exercise (AE) is an effective strategy to reverse recognition memory deficits related to MD. After OR training, NMD rats present increase of NE, which is not observed in any MD groups. One session of physical exercise is not able to change DA or NE hippocampal levels, suggesting that the exercise effects on memory are related to other neural mechanisms.

## Figures and Tables

**Table 1:** Total exploration time in training and tests on the OR task. Data are expressed as the mean ± SD of the total exploration time, in seconds, on OR training and tests (OR; *n* = 8‐10 per group/day; *P* > 0.05; ANOVA). NMD = nonmaternal-deprived rats; MD = maternal-deprived rats; NMD+FAE = nonmaternal-deprived rats submitted to aerobic exercise familiarization; MD+FAE = maternal-deprived rats submitted to aerobic exercise familiarization; NMD+AE = nonmaternal-deprived rats submitted to aerobic exercise familiarization and to an aerobic exercise session; MD+AE = maternal-deprived rats submitted to aerobic exercise familiarization and to an aerobic exercise session.

	NMD	MD	NMD+FAE	MD+FAE	NMD+AE	MD+AE	*P* value
Training (s)	65.10 ± 39.03	44.10 ± 26.78	79.00 ± 22.45	59.40 ± 25.21	66.88 ± 26.19	71.00 ± 31.66	0.1695
Test (24 h)	62.10 ± 29.18	53.29 ± 10.44	86.78 ± 19.85	60.90 ± 35.08	67.25 ± 28.67	78.00 ± 21.63	0.1066
Test (7 d)	41.00 ± 18.93	44.43 ± 21.72	62.22 ± 19.33	42.40 ± 30.18	57.75 ± 22.16	67.00 ± 20.01	0.0539
Test (14 d)	39.60 ± 12.82	35.30 ± 19.69	52.70 ± 21.33	43.40 ± 24.34	55.13 ± 19.53	18.10 ± 20.80	0.0868
Test (21 d)	44.10 ± 12.35	34.00 ± 13.01	45.90 ± 8.54	33.00 ± 14.94	48.33 ± 18.25	47.70 ± 14.80	0.0635

**Table 2:** Maternal deprivation and physical exercise do not alter the anxiety of the animals. Data are expressed as the mean ± SD of the time spent and the total number of entries in the open arms of the EPM. There was no significant difference between the groups on the different days (Kruskal-Wallis test; *n* = 8‐10/group). NMD = nonmaternal-deprived rats; MD = maternal-deprived rats; NMD+FAE = nonmaternal-deprived rats submitted to aerobic exercise familiarization; MD+FAE = maternal-deprived rats submitted to aerobic exercise familiarization; NMD+AE = nonmaternal-deprived rats submitted to aerobic exercise familiarization and to an aerobic exercise session; MD+AE = maternal-deprived rats submitted to aerobic exercise familiarization and to an aerobic exercise session.

	MD	NMD	MD+AE	NMD+AE	MD+FAE	NMD+FAE	*P* value
24 h							
Time in open arms	138.7 ± 19.1	137.5 ± 29.5	145.0 ± 26.6	141.9 ± 26.7	146.0 ± 31.1	148.3 ± 7.7	0.75
Total of entries	16.8 ± 3.8	15.2 ± 5.1	15.9 ± 6.7	17.2 ± 6.4	22.0 ± 6.7	22.80 ± 5.4	0.10
7 days							
Time in open arms	143.7 ± 18.0	139.6 ± 18.3	136.7 ± 22.3	146.0 ± 32.1	151.9 ± 19.1	132.1 ± 9.5	0.21
Total of entries	17.5 ± 5.1	20.0 ± 5.4	18.0 ± 6.4	15.8 ± 5.0	18.7 ± 6.1	20.1 ± 4.1	0.57
14 days							
Time in open arms	133.7 ± 1 6.1	134.3 ± 31.0	142.2 ± 34.9	140.9 ± 27.9	155.0 ± 20.0	148.7 ± 17.2	0.35
Total of entries	12.0 ± 4.2	12.0 ± 3.3	12.4 ± 3.9	13.2 ± 4.2	13.1 ± 4.7	13.5 ± 4.7	0.90
21 days							
Time in open arms	145.0 ± 30.1	149.7 ± 30.8	150.3 ± 41.5	156.9 ± 22.7	148.7 ± 17.7	147.3 ± 28.8	0.95
Total of entries	15.2 ± 2.6	14.0 ± 2.6	17.3 ± 3.5	17.3 ± 2.5	16.1 ± 4.0	15.5 ± 5.2	0.20

## Data Availability

Data will be made available on request.

## Additional Points

*Highlights*. (1) Maternal deprivation causes persistent mnemonic deficits. (2) One aerobic exercise shot reduces mnemonic deficits induced by maternal deprivation. (3) One aerobic exercise shot contributes to memory persistence. (4) Maternal deprivation avoids the hippocampal norepinephrine increase induced by object recognition learning.
